# Long-term intranasal corticosteroids vs. antihistamines and bone health in post-menopausal women with allergic rhinitis: a retrospective cohort study

**DOI:** 10.3389/fmed.2026.1838641

**Published:** 2026-08-10

**Authors:** Xiaofei Jia, Lei Liu, Yanzhong Yang

**Affiliations:** 1 Department of Otolaryngology, The Second Hospital of HeBei Medical University, Shijiazhuang, China; 2 Department of Joint Surgery, The Third Hospital of Hebei Medical University, Shijiazhuang, Hebei, China

**Keywords:** allergic rhinitis, bone mineral density, dose-response, fragility fracture, intranasal corticosteroids, osteoporosis, post-menopausal women

## Abstract

**Background:**

Intranasal corticosteroids (INCS) are first-line therapy for allergic rhinitis (AR), but their long-term skeletal safety in post-menopausal women—who are inherently vulnerable to bone loss—remains inadequately studied despite low systemic bioavailability.

**Methods:**

This retrospective cohort study included 691 post-menopausal women with persistent AR treated from 2015 to 2023. Participants were categorized into INCS (*n* = 198), second-generation antihistamine (sgAH, *n* = 187), combination therapy (*n* = 156), and control (*n* = 150) groups. Primary outcomes were changes in bone mineral density (BMD) *T*-scores and incident osteoporosis. Secondary outcomes included fragility fractures. Multivariable regression analyses were performed with adjustment for confounders, and dose-response relationships were evaluated using defined daily doses.

**Results:**

Over a median follow-up of 4.2 years, INCS users exhibited significantly greater declines in lumbar spine *T*-scores compared to controls, with similar findings at the femoral neck. sgAH users showed no significant bone loss. Dose-response analysis revealed linear trends for greater bone loss with higher cumulative INCS exposure. Incident osteoporosis occurred in 24.2% of INCS users vs. 11.3% of controls. Fragility fracture rates were highest in the INCS group with adjusted HR 3.85 (95% CI = 1.45–10.24, *p* = 0.007) vs. controls. sgAH showed no increased fracture risk (HR 1.13, 95% CI = 0.33–3.89). Subgroup analyses identified heightened risk in women aged ≥65 years and those with BMI < 23 kg/m^2^.

**Conclusions:**

Long-term INCS use in post-menopausal women was associated with accelerated bone loss, increased osteoporosis risk, and higher fracture rates in a dose-dependent manner. These findings raise concerns regarding the assumption of complete INCS skeletal safety in post-menopausal women and suggest the need for enhanced bone monitoring in this population.

## 1 Introduction

Allergic rhinitis (AR) affects over 400 million people worldwide, with a particularly high prevalence among middle-aged and elderly women (1, 2). As a chronic inflammatory condition of the nasal mucosa, AR significantly impairs quality of life through symptoms of sneezing, rhinorrhea, nasal congestion, and pruritus. According to the Allergic Rhinitis and its Impact on Asthma (ARIA) guidelines, intranasal corticosteroids (INCS) are recommended as first-line therapy for moderate-to-severe persistent AR due to their superior efficacy in controlling nasal symptoms compared to antihistamines and leukotriene receptor antagonists (3, 4). Modern INCS formulations, including mometasone furoate, fluticasone propionate, and budesonide, have become the cornerstone of long-term AR management, often requiring continuous use for years or even decades (5).

The systemic safety profile of INCS has generally been considered favorable due to their low bioavailability. Second-generation INCS such as mometasone furoate and fluticasone propionate exhibit systemic bioavailability of less than 1%, theoretically minimizing systemic adverse effects (6). Consequently, these agents are widely regarded as safe for long-term use, with most safety concerns limited to local adverse effects such as nasal irritation, epistaxis, and headache (7). However, this assumption remains inadequately tested in post-menopausal women, a demographic inherently susceptible to accelerated bone loss due to estrogen deficiency-induced disruption of bone remodeling (8). This population exhibits heightened sensitivity to the bone-depleting effects of corticosteroids, as estrogen plays a crucial protective role in maintaining bone mineral density (BMD) by suppressing osteoclast activity and promoting osteoblast survival (9, 10). While systemic corticosteroids are well-established causes of osteoporosis and fragility fractures, even at low doses (2.5–7.5 mg prednisolone equivalent daily), the skeletal safety of INCS in this vulnerable group remains controversial (11).

The existing literature regarding INCS and bone health presents several critical gaps. First, most safety studies have focused on pediatric populations or pre-menopausal adults, with limited representation of post-menopausal women (12). Second, prior investigations have primarily examined short-term outcomes or surrogate markers rather than clinically relevant endpoints such as incident osteoporosis or fragility fractures. Third, while dose-response relationships have been established for oral and inhaled corticosteroids, similar analyses for INCS are lacking. Finally, the comparative bone safety of INCS vs. alternative AR therapies, particularly second-generation antihistamines (sgAH), remains unexplored in this demographic.

Given the widespread and prolonged use of INCS in post-menopausal women and the potential for cumulative systemic exposure to impact bone metabolism, rigorous evaluation of their long-term skeletal effects is warranted. This study aimed to address the aforementioned gaps by: (i) comparing BMD changes, incident osteoporosis, and fracture rates among post-menopausal women with AR receiving INCS, sgAH, combination therapy, or no treatment; (ii) evaluating dose-response relationships between cumulative INCS exposure and skeletal outcomes; and (iii) identifying high-risk subpopulations. To our knowledge, this is the first study to simultaneously examine BMD changes, incident osteoporosis, and fragility fractures in post-menopausal women with AR while incorporating dose-response and comparative safety analyses. We hypothesized that chronic INCS exposure would be associated with greater bone loss compared to untreated controls, with a dose-dependent relationship between cumulative exposure and skeletal outcomes.

## 2 Methods

### 2.1 Study design and setting

This retrospective cohort study was conducted at the Department of Otolaryngology, The Second Hospital of Hebei Medical University, Shijiazhuang, China. The study utilized electronic health records with patient enrollment from January, 2015, to December, 2023, and follow-up through December 31, 2025. This design ensured all enrolled patients had a minimum potential follow-up duration of 2 years. The study protocol received approval from the Institutional Review Board (IRB No. 2026-R336) and was conducted in full accordance with the Declaration of Helsinki. Given the retrospective nature of the study design, the requirement for informed consent was waived by the ethics committee. We adhered to the Strengthening the Reporting of Observational Studies in Epidemiology (STROBE) guidelines for cohort studies to ensure methodological rigor and transparent reporting.

### 2.2 Study population

The study population comprised post-menopausal women diagnosed with persistent allergic rhinitis who presented to our institution during the study period. Inclusion criteria required participants to be female patients aged 50 years or older with confirmed menopausal status, defined as spontaneous amenorrhea for at least 12 consecutive months or bilateral oophorectomy. All participants had to meet the diagnostic criteria for persistent allergic rhinitis according to the Allergic Rhinitis and its Impact on Asthma (ARIA) guidelines and have received continuous pharmacological treatment with intranasal corticosteroids, second-generation oral antihistamines, leukotriene receptor antagonists, or combination therapy for a minimum duration of 2 years. Additionally, eligible participants needed to have both baseline and follow-up dual-energy X-ray absorptiometry measurements available in our electronic database. Patients were excluded if they had any prior systemic corticosteroid use cumulatively exceeding 3 months at any dose, or any use ≥5 mg/day prednisone equivalent for more than 2 weeks, prior or concurrent use of osteoporosis medications, denosumab, selective estrogen receptor modulators, teriparatide, or hormone replacement therapy, or any secondary causes of osteoporosis such as hyperparathyroidism, Cushing's syndrome, chronic kidney disease stage three or higher, or malabsorption syndromes. Other exclusion criteria included active malignancies or history of chemotherapy or radiation therapy, severe hepatic impairment classified as Child-Pugh grade B or C, and inflammatory arthropathies requiring immunosuppressive therapy.

### 2.3 Exposure assessment and group classification

The index date was defined as the date of first prescription for the study medication (INCS, sgAH, or combination therapy) for treated patients, or the date of persistent allergic rhinitis diagnosis for the control group. Follow-up began at the index date and continued until the earliest of: (i) incident osteoporosis diagnosis, (ii) first fragility fracture, (iii) death, (iv) loss to follow-up (>12 months without healthcare contact), or (v) study end (December 31, 2025). Patients enrolled earlier in the study period contributed longer observation periods: those enrolled in 2015–2017 could contribute up to 10 years of follow-up, whereas those enrolled in 2021–2023 contributed 2–4 years.

Patients were categorized into four distinct groups based on their primary long-term allergic rhinitis medication exposure pattern. The first group consisted of patients treated with intranasal corticosteroids including mometasone furoate, fluticasone propionate, or budesonide administered as daily nasal sprays. The second group comprised patients receiving second-generation antihistamines such as cetirizine, loratadine, fexofenadine, or desloratadine at standard daily dosages. The third group included patients on combination therapy involving concurrent use of intranasal corticosteroids and oral antihistamines with or without montelukast. The fourth group served as untreated controls, consisting of post-menopausal women with diagnosed persistent allergic rhinitis (meeting ARIA criteria) and an established index date between January 2015 and December 2023, but no regular pharmacological treatment. Intermittent as-needed medication use of less than 30 days per year was permitted. Controls were followed through December 2025 using identical censoring rules.

Cumulative drug exposure was quantified using the Defined Daily Dose (DDD) methodology over the entire follow-up period and categorized by tertiles: low (< 365 DDD), moderate (365–730 DDD), and high (>730 DDD). These thresholds correspond to approximately 1, 2, and >2 years of continuous daily use. Medication adherence was assessed using the medication possession ratio calculated from prescription refill records, and patients with adherence below 80 percent were excluded to ensure adequate exposure assessment.

### 2.4 Data sources and variable extraction

Covariates extracted for adjustment included demographic characteristics such as age at menopause, body mass index, smoking status, and alcohol consumption; clinical characteristics encompassing allergic rhinitis duration, severity classification according to ARIA guidelines, and comorbid conditions including asthma and atopic dermatitis; concurrent medications with known skeletal effects including proton pump inhibitors (defined as ≥90 days continuous use or recurrent prescriptions within 12 months), thyroid hormones, antidepressants, and anticonvulsants; lifestyle factors such as calcium and vitamin D supplementation and physical activity level; and available laboratory parameters including serum 25-hydroxyvitamin D, calcium, and alkaline phosphatase. Data on race/ethnicity, parental fracture history, prior fragility fractures, and falls history were not consistently available in electronic records and were not included in adjustment.

The primary outcomes included change in BMD T-score calculated as the difference between follow-up and baseline measurements at both lumbar spine and femoral neck sites, and incident osteoporosis defined as T-score equal to or less than negative 2.5 at either skeletal site during follow-up in patients with baseline T-scores above this threshold. Secondary outcomes comprised fragility fractures confirmed radiologically as low-trauma fractures resulting from falls no greater than standing height, specifically including vertebral, hip, wrist, and humerus fractures, as well as time to first fragility fracture.

### 2.5 BMD assessment

BMD was measured using dual-energy X-ray absorptiometry systems including Hologic Discovery Wi or Lunar iDXA at the lumbar spine from first to fourth vertebrae and femoral neck according to standardized protocols established by the International Society for Clinical Densitometry. Rigorous quality assurance procedures were implemented including daily phantom scans and cross-calibration between different devices to ensure measurement consistency. The precision error for both measurement sites was maintained below 1.5 percent. *T*-scores were calculated using manufacturer-specific reference databases appropriate for the study population ethnicity.

### 2.6 Statistical analysis

Sample size estimation was based on prior studies demonstrating a 0.3 to 0.5 standard deviation difference in BMD change between corticosteroid-exposed and unexposed post-menopausal women, indicating that 120 patients per group would provide 80 percent statistical power to detect a 0.3 difference in *T*-score change at a two-tailed significance level of 0.050. Continuous variables were summarized using mean and standard deviation or median and interquartile range depending on distributional characteristics, while categorical variables were presented as frequencies and percentages. Baseline characteristics across treatment groups were compared using analysis of variance, Kruskal–Wallis tests, or chi-square tests as appropriate for variable type and distribution.

Between-group comparisons of continuous outcomes were first assessed using analysis of variance (ANOVA) for unadjusted overall differences, followed by multivariable linear regression for adjusted pairwise comparisons with the control group as reference. The primary analysis employed multivariable linear regression to evaluate the association between treatment group assignment and change in *T*-score, with three hierarchical models constructed to demonstrate the impact of confounding control: Model 0 (unadjusted crude association), Model 1 (adjusted for age, BMI, menopausal duration, and baseline *T*-score), and Model 2 (additionally adjusted for AR severity, concurrent bone-depleting medications, calcium/vitamin D supplementation, and physical activity level). Secondary analyses utilized logistic regression to estimate odds ratios for incident osteoporosis with the same covariate adjustment as Model 2, and Cox proportional hazards regression to calculate hazard ratios for time to first fragility fracture with corresponding Kaplan–Meier survival curves for visualization. Competing risks analysis was additionally performed for fracture outcomes considering mortality as a competing event.

Sensitivity analyses included propensity score matching using one-to-one nearest neighbor algorithm with a caliper width of 0.2 to achieve balance in baseline characteristics between intranasal corticosteroid users and controls, stratified analysis by cumulative defined daily dose categories for intranasal corticosteroid users to assess dose-response relationships, subgroup analysis excluding patients with baseline osteopenia to evaluate effects specifically on normal bone mass, and landmark analysis restricting to patients with at least 3 years of follow-up to ensure adequate exposure duration.

Missing data were handled using multiple imputation by chained equations (*m* = 20 imputations) under the assumption of missing at random, with sensitivity comparisons between complete-case and imputed datasets. Missing data proportions were: serum 25-hydroxyvitamin D 12.3%, physical activity level 8.7%, alcohol consumption 6.2%, and alkaline phosphatase 4.1%; all other covariates had less than 3% missing. All statistical analyses were conducted using R software version 4.3.0 and Stata version 17.0, with statistical significance determined at a two-tailed *p*-value threshold of 0.05.

## 3 Results

### 3.1 Study population

**Figure 1:**
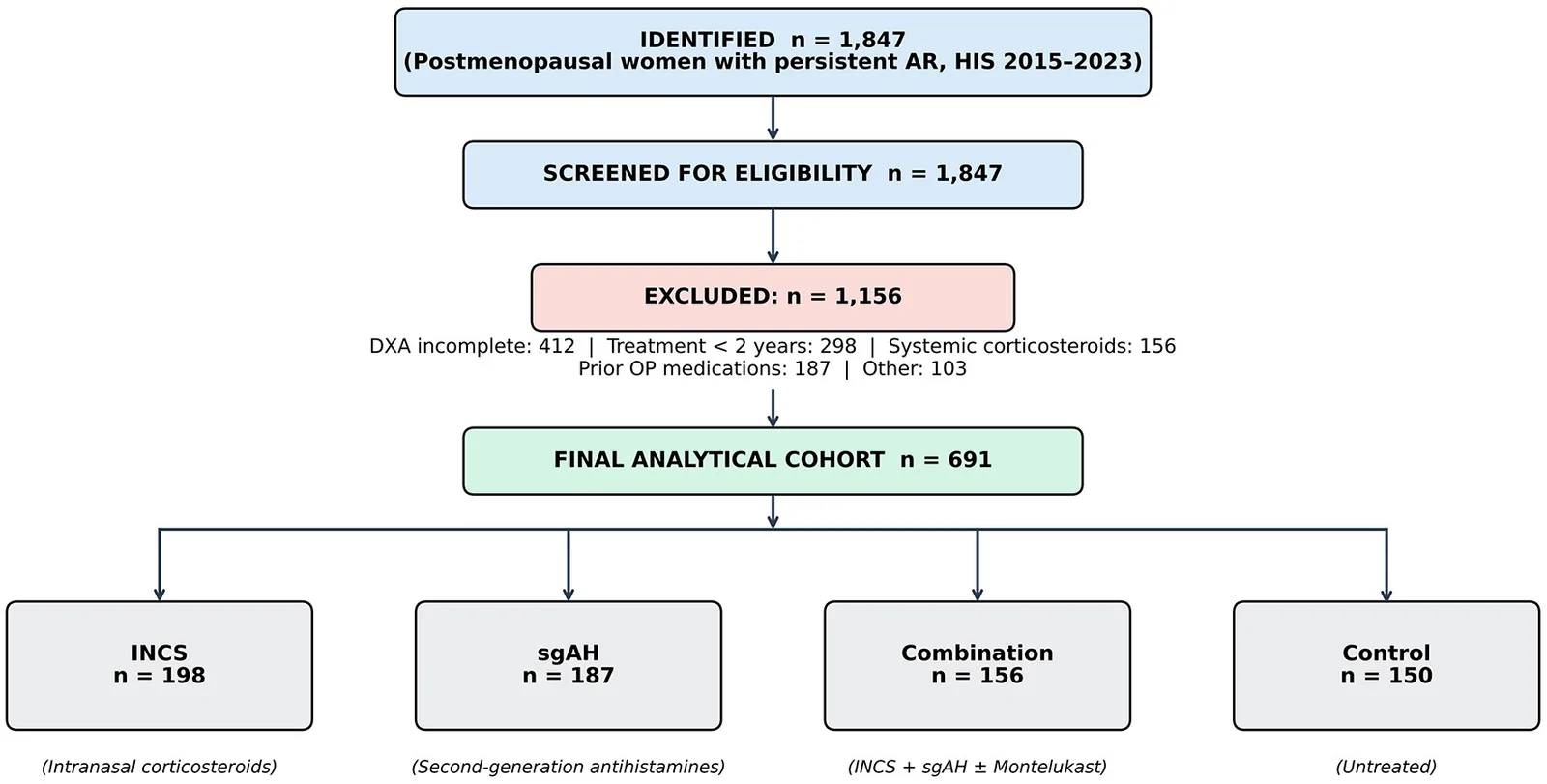
Study flow diagram showing patient selection process from initial identification to final analysis cohort. INCS, intranasal corticosteroids; sgAH, second-generation antihistamines; AR, allergic rhinitis; DXA, dual-energy X-ray absorptiometry; MPR, medication possession ratio; HIS, hospital information system.

A total of 1,847 post-menopausal women with persistent allergic rhinitis were initially identified from the Hospital Information System between January 2015 and December 2023. After applying inclusion and exclusion criteria, 1,156 patients were excluded due to incomplete dual-energy X-ray absorptiometry data (*n* = 412), insufficient treatment duration (*n* = 298), concurrent use of systemic corticosteroids (*n* = 156), prior osteoporosis medication use (*n* = 187), or other exclusion criteria (*n* = 103). The final analytical cohort comprised 691 patients, including 198 in the intranasal corticosteroid group, 187 in the second-generation antihistamine group, 156 in the combination therapy group, and 150 in the age-matched healthy control group (Figure 1).

Follow-up extended through December 2025. Median follow-up was 4.2 years (IQR = 3.1–5.8). Baseline characteristics were comparable across groups, with mean age 60.5–62.4 years and baseline lumbar spine *T*-scores ranging from −1.12 to −1.28 (*p* = 0.456). The prevalence of baseline osteopenia was 38.5%−45.5% across groups without significant difference (*p* = 0.512). INCS users had median cumulative defined daily dose of 587 (IQR = 412–823), with 34.3%, 41.4%, and 24.3% in low, moderate, and high exposure categories, respectively. Total follow-up accumulated 3,055 person-years across the cohort: 840 PY in the INCS group, 780 PY in the sgAH group, 740 PY in the combination group, and 735 PY in the control group. The combination and control groups had slightly longer mean follow-up due to higher proportion of early enrollees with longer observation periods. Baseline demographic and clinical characteristics are presented in Table 1.

**Table 1:** Baseline characteristics of study population.

Characteristic	INCS	sgAH	Combination	Control	*p*
	(*n* = 198)	(*n* = 187)	(*n* = 156)	(*n* = 150)	
Age, years	62.4 ± 7.8	61.8 ± 8.1	60.5 ± 7.5	61.2 ± 7.9	0.234
BMI, kg/m^2^	24.6 ± 3.4	23.9 ± 3.1	24.3 ± 3.3	24.1 ± 3.2	0.187
Menopausal duration, years	11.2 (6.3–15.7)	10.8 (5.9–15.2)	9.6 (5.2–14.1)	10.5 (6.1–14.8)	0.312
Baseline LS *T*-score	−1.18 ± 0.92	−1.12 ± 0.89	−1.28 ± 0.95	−1.15 ± 0.91	0.456
Baseline FN *T*-score	−1.52 ± 0.78	−1.42 ± 0.81	−1.48 ± 0.83	−1.35 ± 0.79	0.389
Baseline osteopenia, *n* (%)	84 (42.3)	72 (38.5)	71 (45.5)	60 (40.0)	0.512
Concurrent asthma, *n* (%)	46 (23.2)	31 (16.6)	52 (33.3)	18 (12.0)	< 0.001
Calcium/vitamin D supplementation, *n* (%)	89 (44.9)	78 (41.7)	68 (43.6)	62 (41.3)	0.876
AR duration, years	8.2 ± 4.5	7.9 ± 4.2	8.5 ± 4.8	7.6 ± 4.0	0.312
AR severity (moderate–severe), *n* (%)	156 (78.8)	142 (75.9)	138 (88.5)	98 (65.3)	< 0.001
Prior fracture, *n* (%)	12 (6.1)	9 (4.8)	11 (7.1)	8 (5.3)	0.789
Parental hip fracture, *n* (%)	23 (11.6)	19 (10.2)	22 (14.1)	15 (10.0)	0.654
Falls in past year, *n* (%)	31 (15.7)	26 (13.9)	29 (18.6)	19 (12.7)	0.456
PPI use, *n* (%)	46 (23.2)	31 (16.6)	52 (33.3)	18 (12.0)	< 0.001
Thyroid hormone use, *n* (%)	18 (9.1)	15 (8.0)	14 (9.0)	11 (7.3)	0.876
Physical activity (moderate-high), *n* (%)	89 (44.9)	78 (41.7)	68 (43.6)	62 (41.3)	0.876
Serum 25(OH)D, nmol/L	52.3 ± 18.6	54.1 ± 19.2	50.8 ± 17.9	53.5 ± 18.4	0.423

Data were presented as mean ± SD, median (IQR), or n (%). BMI, body mass index; LS, lumbar spine; FN, femoral neck; INCS, intranasal corticosteroids; sgAH, second-generation antihistamines.

### 3.2 BMD changes

All groups demonstrated expected post-menopausal bone loss, with INCS users showing the greatest decline (Table 2). Between-group differences were evaluated using analysis of variance for unadjusted comparisons and multivariable linear regression for adjusted pairwise comparisons, with the control group as reference. In fully adjusted analysis, INCS use was associated with significantly greater lumbar spine *T*-score decline compared to controls (adjusted mean difference −0.13, 95% CI = −0.24 to −0.02, *p* = 0.019), while sgAH showed no significant difference (−0.04, 95% CI −0.15 to 0.07, *p* = 0.478). Femoral neck results were consistent (INCS: −0.16, 95% CI −0.28 to −0.04, *p* = 0.011; sgAH: −0.05, 95% CI −0.16 to 0.06, *p* = 0.384).

**Table 2:** Changes in bone mineral density (BMD) *T*-scores by treatment group.

Outcome	INCS (*n* = 198)	sgAH (*n* = 187)	Combination (*n* = 156)	Control (*n* = 150)	*p* ^*^
Lumbar spine ΔT-score					
Unadjusted	−0.38 (−0.45, −0.31)	−0.26 (−0.33, −0.19)	−0.34 (−0.42, −0.26)	−0.22 (−0.29, −0.15)	0.008
Model 1†	−0.16 (−0.27, −0.05)	−0.06 (−0.17, 0.05)	−0.12 (−0.24, −0.01)	Reference	0.031
Model 2‡	−0.13 (−0.24, −0.02)	−0.04 (−0.15, 0.07)	−0.10 (−0.21, 0.01)	Reference	0.048
Femoral neck ΔT-score					
Unadjusted	−0.42 (−0.50, −0.34)	−0.28 (−0.36, −0.20)	−0.36 (−0.45, −0.27)	−0.24 (−0.32, −0.16)	0.003
Model 1†	−0.18 (−0.30, −0.06)	−0.05 (−0.17, 0.07)	−0.11 (−0.23, 0.01)	Reference	0.022
Model 2‡	−0.16 (−0.28, −0.04)	−0.05 (−0.16, 0.06)	−0.09 (−0.21, 0.03)	Reference	0.041

^*^Overall ANOVA p-value for unadjusted comparison across all four groups. Pairwise comparisons were performed using multivariable linear regression with control as reference.

^†^Model 1: adjusted for age, BMI, menopausal duration, and baseline *T*-score.

^‡^Model 2: additionally adjusted for AR severity, concurrent bone-depleting medications, calcium/vitamin D supplementation, and physical activity.

**Figure 2:**
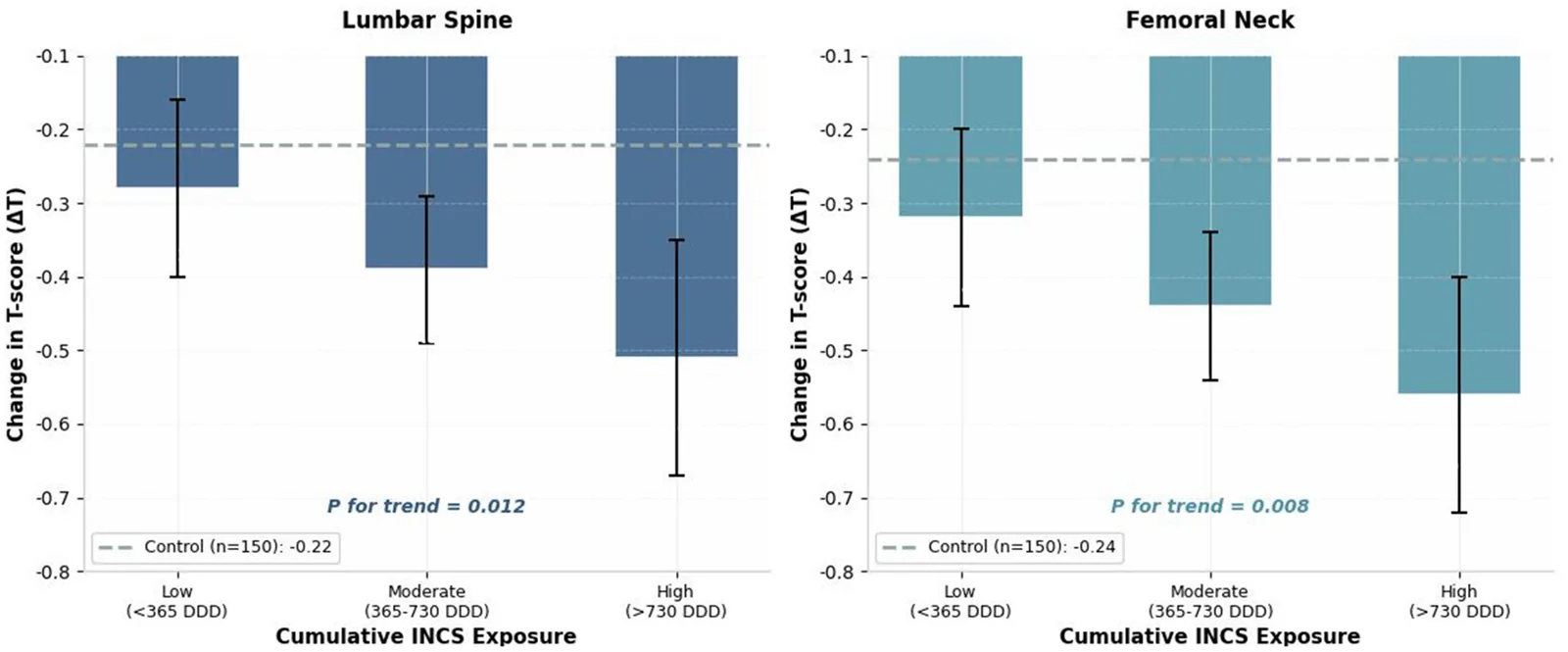
Dose-response relationship between cumulative intranasal corticosteroid exposure and changes in BMD *T*-scores. Low exposure: < 365 DDD; moderate: 365–730 DDD; high: >730 DDD. Error bars represent 95% confidence intervals. *P*-values for linear trend: lumbar spine *p* = 0.012, femoral neck *p* = 0.008.

Dose-response analysis within the intranasal corticosteroid group revealed a linear trend of greater bone loss with higher cumulative exposure over the entire follow-up period. Patients in the low exposure category (< 365 DDD) showed lumbar spine *T*-score change of −0.28 (SE = 0.06), moderate exposure (365–730 DDD) showed −0.39 (SE = 0.05), and high exposure (>730 DDD) showed −0.51 (SE = 0.08; *p* for trend = 0.012). Similarly, femoral neck *T*-score changes demonstrated a dose-dependent pattern with changes of −0.32, −0.44, and −0.56 for low, moderate, and high exposure categories respectively (*p* for trend = 0.008, Figure 2).

### 3.3 Incident osteoporosis

During the follow-up period, 127 patients (18.4%) developed incident osteoporosis defined as *T*-score declining to −2.5 or below at either the lumbar spine or femoral neck. The incidence varied significantly across treatment groups, occurring in 48 of 198 intranasal corticosteroid users (24.2%), 24 of 187 antihistamine users (12.8%), 38 of 156 combination therapy patients (24.4%), and 17 of 150 controls (11.3%). The overall difference was statistically significant (*p* = 0.002). In multivariable logistic regression adjusting for all potential confounders, intranasal corticosteroid use was associated with significantly increased odds of incident osteoporosis compared to controls (adjusted odds ratio 2.48, 95% confidence interval 1.32 to 4.67, *p* = 0.005). Combination therapy also showed elevated risk (adjusted odds ratio 2.35, 95% confidence interval 1.21 to 4.56, *p* = 0.011), while antihistamine use was not significantly associated with increased risk (adjusted odds ratio 1.18, 95% confidence interval 0.58 to 2.41, *p* = 0.648). When analyzed by cumulative dose, high-exposure intranasal corticosteroid users demonstrated substantially elevated risk (adjusted odds ratio 3.62, 95% confidence interval 1.58 to 8.29, *p* = 0.002) compared to controls, while moderate exposure showed borderline significance (adjusted odds ratio 2.14, 95% confidence interval 1.02 to 4.48, *p* = 0.044) and low exposure did not differ significantly from controls (adjusted odds ratio 1.68, 95% confidence interval 0.72 to 3.92, *p* = 0.234). The population attributable fraction for incident osteoporosis due to intranasal corticosteroid use was estimated at 18.7% (95% confidence interval 7.2 to 30.2%, Table 3).

**Table 3:** Incidence of osteoporosis and fragility fractures.

Outcome	INCS (*n* = 198)	sgAH (*n* = 187)	Combination (*n* = 156)	Control (*n* = 150)	Adjusted OR/HR (95% CI)^*^	*P*
Incident osteoporosis, *n* (%)	48 (24.2)	24 (12.8)	38 (24.4)	17 (11.3)		0.002†
vs. control					2.48 (1.32–4.67)‡	0.005
vs. control					1.18 (0.58–2.41)^**§**^	0.648
vs. control					2.35 (1.21–4.56)^**||**^	0.011
Fragility fractures, *n* (rate/1,000 PY)	22 (26.2)	6 (7.7)	14 (18.9)	5 (6.8)		
vs. control					3.85 (1.45–10.24)‡	0.007
vs. control					1.13 (0.33–3.89)^**§**^	0.836
vs. control					2.78 (0.98–7.89)^**||**^	0.055

^*^PY, person-years. Adjusted for age, BMI, menopausal duration, baseline *T*-score, AR severity, concurrent bone-depleting medications, calcium/vitamin D supplementation, and physical activity.

^†^Overall chi-square p-value.

^‡^INCS vs. control.

^**§**^sgAH vs. control.

^**||**^Combination vs. control.

### 3.4 Fragility fractures

**Figure 3:**
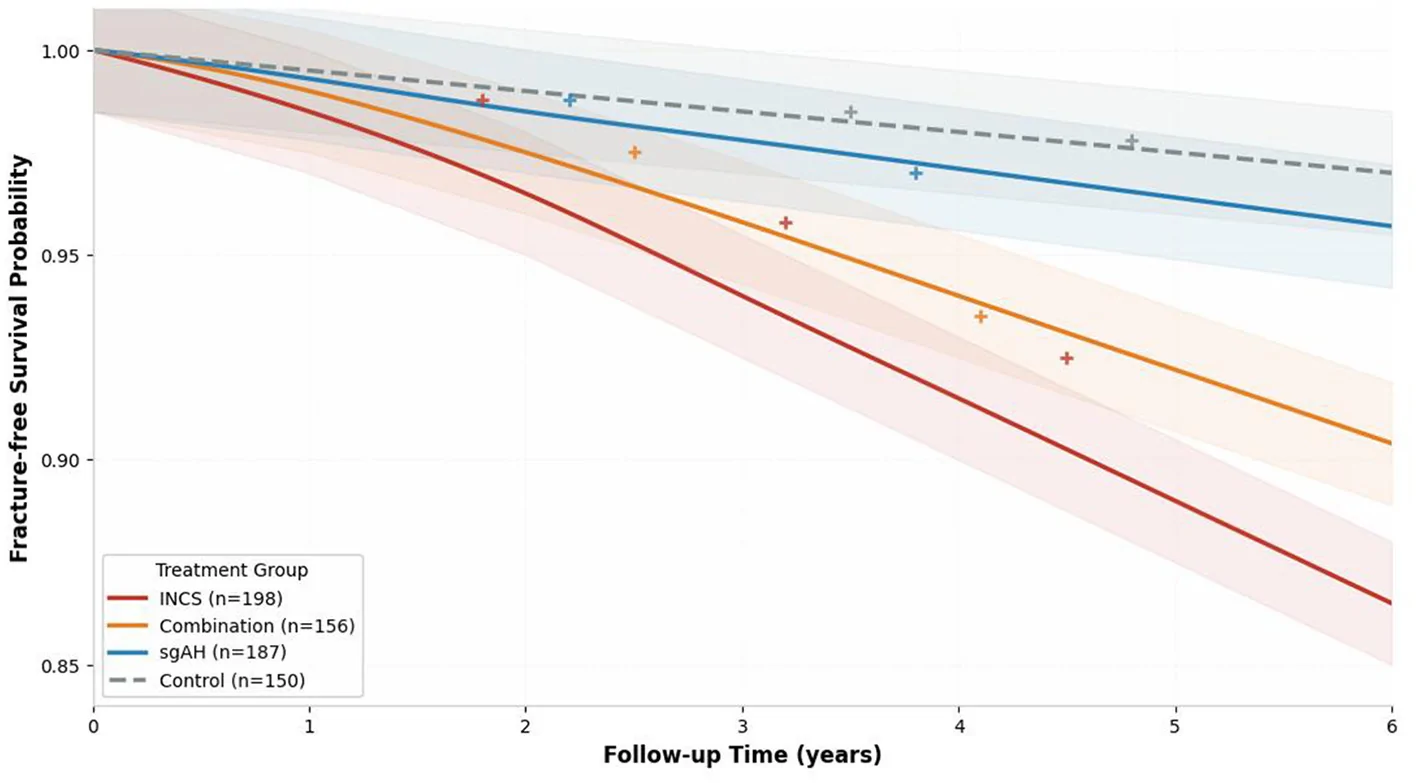
Kaplan–Meier curves showing cumulative incidence of first fragility fracture by treatment group. Log-rank test *p* = 0.014. INCS, intranasal corticosteroids; sgAH, second-generation antihistamines; Combo, combination therapy.

Forty-three fragility fractures occurred (incidence 15.6/1,000 person-years), with distribution as follows: vertebral 18, wrist 12, hip eight, humerus five. Incidence rates were highest in INCS users (26.2/1,000 PY), followed by combination therapy (18.9/1,000 PY), sgAH (7.7/1,000 PY), and controls (6.8/1,000 PY). The dose-response gradient across treatment intensities is consistent with a potentially causal association between corticosteroid exposure and fracture risk. Kaplan-Meier analysis demonstrated significant differences in time to first fracture (log-rank *p* = 0.014, Figure 3). In adjusted Cox regression, INCS use was associated with increased fracture hazard (adjusted HR 3.85, 95% CI = 1.45–10.24, *p* = 0.007), while sgAH showed no association (adjusted HR 1.13, 95% CI = 0.33–3.89, *p* = 0.836). Competing risks analysis considering mortality as a competing event yielded results consistent with the primary Cox analysis (subdistribution HR 3.72, 95% CI = 1.38–10.05 for INCS vs. control).

### 3.5 Sensitivity analyses

Propensity score-matched analysis (142 pairs) confirmed robustness of primary findings (Table 4), with INCS use remaining associated with greater lumbar spine *T*-score decline (mean difference −0.14, 95% CI −0.27 to −0.01, *p* = 0.038) and incident osteoporosis (adjusted OR 2.19, 95% CI = 1.08–4.44, *p* = 0.030). Exclusion of baseline osteopenia patients (*n* = 389) yielded consistent results for bone loss progression (adjusted OR 1.89, 95% CI = 1.05–3.41, *p* = 0.034). Landmark analysis (≥3 years follow-up, *n* = 412) and complete-case analysis showed minimal variation in effect estimates (< 5% relative change).

**Table 4:** Sensitivity analyses for primary outcomes.

Analysis	Lumbar spine ΔT-score		Incident osteoporosis	
	Mean difference (95% CI)	*p*-value	Adjusted OR (95% CI)	*p*
Primary analysis	−0.13 (−0.24, −0.02)	0.019	2.48 (1.32, 4.67)	0.005
Propensity score matched	−0.14 (−0.27, −0.01)	0.038	2.19 (1.08, 4.44)	0.030
Excluding baseline osteopenia	−0.11 (−0.22, −0.00)	0.046	1.89 (1.05, 3.41)	0.034
Landmark (≥3 years)	−0.15 (−0.28, −0.02)	0.022	2.71 (1.31, 5.61)	0.007
Complete case	−0.14 (−0.25, −0.03)	0.015	2.62 (1.37, 5.01)	0.004

All models adjusted for age, BMI, menopausal duration, baseline *T*-score, AR severity, concurrent bone-depleting medications, calcium/vitamin D supplementation, and physical activity.

### 3.6 Subgroup analyses

**Figure 4:**
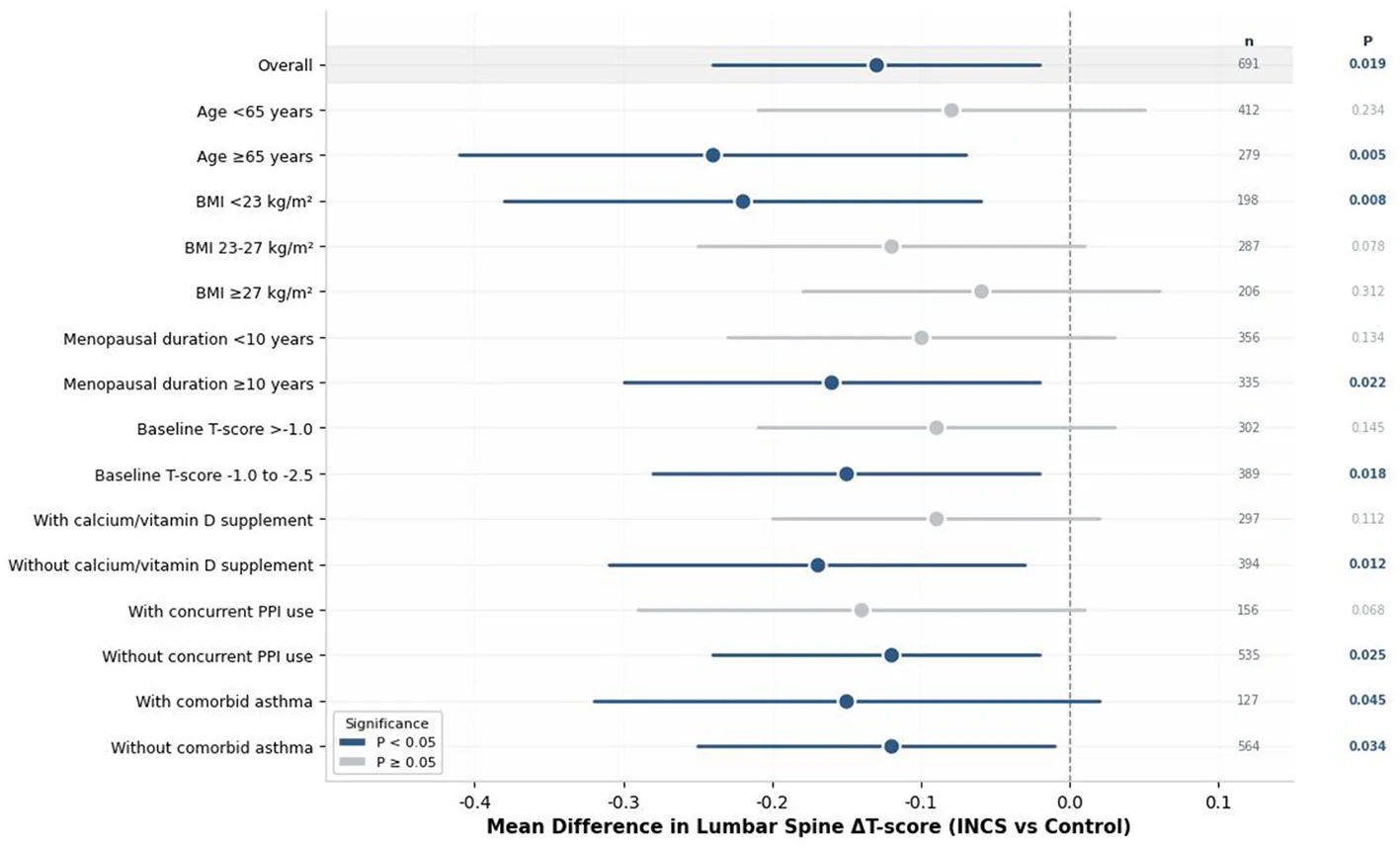
Exploratory subgroup analysis of intranasal corticosteroid effects on lumbar spine *T*-score changes. Effect modification tested by interaction terms in fully adjusted models. *P*-values for interaction are unadjusted for multiple testing.

Exploratory subgroup analyses were conducted to identify potential effect modification by baseline characteristics and were not adjusted for multiple comparisons (Figure 4). Significant interactions were observed between intranasal corticosteroid use and age, with patients aged 65 years or older demonstrating more pronounced bone loss (interaction *p* = 0.043). In this older subgroup, intranasal corticosteroid use was associated with adjusted mean difference in lumbar spine *T*-score of −0.24 (95% confidence interval −0.41 to −0.07) compared to −0.08 (95% confidence interval −0.21 to 0.05) in younger patients. Similarly, significant interaction was observed with body mass index, where lean patients (body mass index less than 23 kg/m^2^) showed greater vulnerability to corticosteroid-induced bone loss (interaction *p* = 0.038). Concurrent use of proton pump inhibitors, which may independently affect bone metabolism, did not significantly modify the association between intranasal corticosteroids and bone outcomes (interaction *p* = 0.672 for *T*-score change, *p* = 0.584 for incident osteoporosis). Calcium and vitamin D supplementation appeared to attenuate but not eliminate the adverse effects, with supplemented patients showing smaller but still significant differences in bone loss compared to unsupplemented intranasal corticosteroid users (interaction *p* = 0.089). Presence of comorbid asthma, which might indicate systemic inflammation or alternative corticosteroid exposure, did not significantly interact with treatment effects (interaction *p* = 0.456).

## 4 Discussion

This retrospective cohort study provides evidence suggesting that long-term INCS use in post-menopausal women with allergic rhinitis was associated with accelerated bone loss, increased risk of incident osteoporosis, and higher fragility fracture rates compared to untreated controls. Specifically, we observed that INCS users experienced significantly greater declines in both lumbar spine and femoral neck *T*-scores (adjusted mean differences of −0.13 and −0.16, respectively), with a clear dose-response relationship indicating that higher cumulative exposure (>730 defined daily doses) nearly doubled the magnitude of bone loss compared to lower exposure categories. These findings raise concerns regarding the prevailing clinical assumption that modern INCS formulations, despite their low systemic bioavailability (< 1%), are entirely devoid of clinically significant skeletal effects in vulnerable populations.

The magnitude of bone loss observed in our INCS users, while modest compared to oral corticosteroid therapy (which typically induces annual *T*-score declines of 0.5–1.0), remains clinically meaningful within the context of post-menopausal bone physiology (13, 14). Our results appear to contradict several short-term randomized controlled trials demonstrating no significant changes in BMD with 1-year INCS use in pre-menopausal or mixed populations (15). However, this discrepancy is biologically plausible and likely reflects three critical distinctions: first, the extended follow-up duration in our study (median 4.2 years vs. ≤ 12 months in randomized controlled trials) allows detection of cumulative effects that short-term exposure cannot reveal (16, 17); second, our exclusive focus on post-menopausal women captures a demographic with heightened skeletal vulnerability due to estrogen deficiency-induced disruption of bone remodeling balance (18, 19); and third, the dose-response gradient we identified suggests that only sustained high-dose exposure exceeds the threshold for clinical detectability, a scenario rarely tested in controlled trials with limited follow-up.

Importantly, our findings align with emerging real-world evidence from large administrative databases. A Canadian population-based registry study reported that older women with high adherence to inhaled corticosteroid use (≥720 days) had modestly lower hip BMD (−0.14 *T*-score) and accelerated bone loss (−0.02 SD/year) compared to non-users, despite no significant increase in fracture risk (19–21). Similarly, a meta-analysis of observational studies by Loke et al. found that inhaled corticosteroids were associated with a modest but statistically significant increased risk of fractures in COPD patients (OR 1.21, 95% CI = 1.12–1.32), with a dose-response relationship indicating a 9% increased fracture risk per 500 μg increase in beclomethasone equivalents (22). The consistency across geographically diverse populations strengthens the generalizability of our observations and suggests that previous null findings from short-term randomized trials may have resulted from insufficient follow-up duration or inclusion of younger, bone-resilient populations rather than true absence of effect (23).

The pathophysiological mechanisms underlying our observations likely involve cumulative systemic glucocorticoid exposure achieving threshold concentrations sufficient to activate bone-depleting cellular pathways, albeit at reduced magnitude compared to oral or inhaled formulations (24). While second-generation INCS such as mometasone and fluticasone exhibit systemic bioavailability below 1% (25), analogous evidence from inhaled corticosteroids (ICS)—which share similarly low bioavailability profiles—demonstrates that chronic exposure can achieve clinically relevant skeletal effects. A Canadian registry study of older women found that high-adherence ICS users (≥720 days) exhibited lower hip BMD and accelerated bone loss compared to non-users (20), and a meta-analysis reported a 21% increased fracture risk with ICS use (OR 1.21, 95% CI = 1.12–1.32) with a dose-dependent gradient of 9% increased risk per 500 μg beclomethasone equivalent (22). These data establish that low systemic bioavailability does not preclude cumulative bone toxicity. In the context of INCS, several factors may further amplify systemic exposure in chronic users. First, persistent nasal mucosal inflammation in allergic rhinitis may compromise mucosal integrity, potentially increasing drug absorption through disrupted epithelial barriers. Second, long-term daily administration results in cumulative exposure that may overwhelm hepatic first-pass metabolism over years, particularly in post-menopausal women who often exhibit age-related declines in hepatic clearance capacity (26, 27).

At the cellular level, glucocorticoids exert multifaceted detrimental effects on bone metabolism that parallel the established pathophysiology of glucocorticoid-induced osteoporosis (24). These include suppression of osteoblast proliferation and differentiation through inhibition of Wnt/β-catenin signaling, promotion of osteoblast and osteocyte apoptosis, and enhancement of osteoclast lifespan through upregulation of receptor activator of nuclear factor-κB ligand (RANKL) combined with downregulation of osteoprotegerin (28, 29). Importantly, recent evidence indicates that glucocorticoid-induced osteocyte apoptosis reduces mechanosensory function and disrupts the osteocyte-lacunar-canalicular network essential for targeted bone remodeling (28). Our observation that bone loss occurred at both lumbar spine (trabecular bone-rich) and femoral neck (cortical-trabecular composite) sites suggests that INCS exposure affects both bone compartments, consistent with systemic glucocorticoid activity rather than localized mechanical factors (30).

The absence of significant bone loss in the sgAH group carries important clinical implications and serves as a negative control supporting the specificity of our INCS findings. Unlike first-generation antihistamines, which possess anticholinergic properties associated with increased fall and fracture risk in elderly populations (OR 2.03, 95% CI = 1.49–2.76) (31), modern sgAH such as cetirizine and fexofenadine lack significant anticholinergic activity (32) and do not directly influence osteoblast or osteoclast function. Our results align with observational studies suggesting neutral effects of antihistamines on BMD (33), indicating that the bone loss observed in INCS users cannot be attributed to allergic rhinitis itself or shared disease characteristics between treatment groups.

Our subgroup analyses revealed significant effect modification by age and body mass index, identifying specific populations at heightened risk for INCS-related bone loss. Patients aged ≥65 years exhibited nearly three-fold greater *T*-score declines compared to younger post-menopausal women (adjusted mean difference −0.24 vs. −0.08), likely reflecting the convergence of multiple risk factors: lower residual estrogen levels, reduced baseline bone formation capacity, and age-related vitamin D deficiency (34). Similarly, lean patients (BMI < 23 kg/m^2^) demonstrated heightened vulnerability, consistent with established observations that low body weight amplifies systemic corticosteroid bone toxicity (35). The interaction between calcium/vitamin D supplementation and INCS effects (*p* = 0.089) suggests a trend toward attenuation of bone loss in supplemented users, though the effect remained statistically significant even among supplemented individuals (36, 37). This finding implies that nutritional optimization serves as an adjunctive rather than primary preventive strategy in this population. Clinicians should not assume that calcium and vitamin D supplementation alone will neutralize the skeletal risks of chronic INCS exposure in high-risk post-menopausal women.

Current ARIA guidelines recommend INCS as first-line therapy for moderate-to-severe persistent AR without specific consideration of bone safety in post-menopausal women. Our data suggest that this population—particularly those aged ≥65 years or with BMI < 23 kg/m^2^—may warrant enhanced monitoring, including baseline dual-energy X-ray absorptiometry assessment and periodic rescreening at 2–3 year intervals when chronic INCS therapy is anticipated. The risk-benefit calculus of AR pharmacotherapy should be individualized based on skeletal risk profiles. For post-menopausal women with established osteopenia, multiple osteoporosis risk factors, or high baseline fracture probability, initiating sgAH therapy as first-line treatment with step-up to INCS only if symptom control proves inadequate may represent a reasonable de-escalation strategy, provided disease severity permits. For those requiring INCS, use of the lowest effective dose, regular bone density monitoring, and concurrent optimization of calcium/vitamin D status and physical activity should become standard of care.

While residual confounding by unmeasured disease severity or systemic inflammation cannot be fully excluded in this observational design, several observations support the specificity of our findings for intranasal corticosteroid exposure. First, multivariable adjustment for ARIA severity classification and concurrent asthma diagnosis did not materially attenuate the association between INCS use and bone loss. Second, the sgAH group, which treated patients with comparable AR severity (75.9% moderate–severe vs. 78.8% in the INCS group), showed no significant bone loss, serving as a pharmacological negative control. Third, the presence of a graded dose-response relationship between cumulative INCS exposure and bone loss is inconsistent with a pure confounding explanation.

Several limitations warrant cautious interpretation of our findings and highlight directions for future research. First, the retrospective design introduces potential for residual confounding. Patients requiring long-term INCS likely represent a more severe disease population with different health-seeking behaviors and unmeasured comorbidities than untreated controls, and this fundamental limitation cannot be fully corrected through statistical adjustment. However, the absence of significant bone loss in the sgAH group—who treated patients with comparable AR severity—suggests that confounding by indication alone is unlikely to fully explain our findings. However, the absence of bone loss in the sgAH group—which treats similar symptomatic AR—suggests that confounding by disease severity alone is unlikely to explain our findings. Second, 62.6% of initially identified patients were excluded, primarily due to missing DXA data. While DXA measurements are required for our primary outcomes, we acknowledge that women who underwent screening may differ systematically from those who did not. Complete-case analysis (Table 4) yielded consistent effect estimates (< 5% relative change from primary analysis), supporting internal validity. Generalizability to the broader population of post-menopausal women with allergic rhinitis should be interpreted with caution. Third, we lacked detailed data on specific INCS formulations (mometasone furoate vs. fluticasone propionate vs. budesonide), which possess distinct pharmacokinetic profiles including varying degrees of systemic absorption and hepatic clearance. Future studies should determine whether bone toxicity differs by specific agent, as this would inform safer prescribing practices within the INCS class. Fourth, the modest absolute number of fracture events (*n* = 43) limited our statistical power to detect differences in specific fracture types (e.g., hip vs. vertebral) or to perform extensive subgroup analyses for fracture outcomes. The resulting wide confidence intervals for some hazard ratios (e.g., 1.45–10.24 for INCS vs. control) reflect this imprecision and should be interpreted as suggesting a clinically meaningful association whose exact magnitude requires confirmation in larger cohorts. Fifth, our reliance on electronic health records precluded assessment of adherence to calcium/vitamin D supplementation, dietary calcium intake, or over-the-counter supplement use, which may have influenced bone outcomes. Similarly, we lacked data on objective measures of systemic corticosteroid exposure such as serum cortisol suppression or urinary cortisol metabolites, which would strengthen causal inference regarding systemic absorption. Sixth, we were unable to analyze specific INCS formulations separately due to limited sample sizes for individual agents. Mometasone furoate, fluticasone propionate, and budesonide have distinct pharmacokinetic profiles and systemic absorption characteristics, and pooling them may obscure differential skeletal effects. Future studies with larger cohorts should determine whether bone toxicity varies by specific agent, which would inform safer prescribing within the INCS class. Finally, drug exposure was estimated from prescription records and defined daily doses, which may not reflect actual use or administration technique. We excluded patients with medication possession ratio below 80% to improve adherence assessment. Non-differential misclassification would bias effects toward the null, suggesting our estimates may be conservative.

Future research should confirm these associations in prospective cohorts with larger fracture event numbers and directly compare bone outcomes between INCS and sgAH in randomized trials specifically designed for post-menopausal populations. Determining whether specific INCS agents differ in skeletal toxicity—and whether antihistamine co-therapy mitigates bone loss—would inform safer prescribing strategies within this drug class.

This study makes several contributions to the literature. To our knowledge, it is the first to simultaneously examine BMD changes, incident osteoporosis, and fragility fractures in post-menopausal women with allergic rhinitis, while incorporating dose-response and comparative safety analyses against antihistamines. The identification of a dose-dependent relationship and high-risk subgroups (age ≥65 years, BMI < 23 kg/m^2^) provides actionable guidance for clinicians managing long-term AR therapy in this vulnerable population. The plausibility of our findings is supported by the modest effect sizes (consistent with low systemic bioavailability), the linear exposure-response gradient, and the specificity of effects to corticosteroid-exposed groups.

## 5 Conclusion

In conclusion, this study suggests that long-term INCS use in post-menopausal women was associated with accelerated bone loss, increased osteoporosis risk, and higher fracture rates in a dose-dependent manner. These findings raise concerns about the prevailing assumption of complete INCS skeletal safety in this population and support consideration of enhanced bone monitoring, particularly in women with additional risk factors for osteoporosis. Clinicians should consider baseline bone density assessment, periodic monitoring, and potentially alternative therapeutic strategies for post-menopausal women requiring long-term AR therapy, particularly those with additional risk factors for osteoporosis.

## Data Availability

The original contributions presented in the study are included in the article/supplementary material, further inquiries can be directed to the corresponding author.

## References

[1] Nur Husna SM Tan HT Md Shukri N Mohd Ashari NS Wong KK. Allergic rhinitis: a clinical and pathophysiological overview. Front Med (Lausanne). (2022) 9:874114. doi: 10.3389/fmed.2022.874114 35463011 PMC9021509

[2] Cazzoletti L Ferrari M Olivieri M Verlato G Antonicelli L Bono R . The gender, age and risk factor distribution differs in self-reported allergic and non-allergic rhinitis: a cross-sectional population-based study. Allergy Asthma Clin Immunol. (2015) 11:36. doi: 10.1186/s13223-015-0101-1 26640494 PMC4669616

[3] Bousquet J Schünemann HJ Togias A Bachert C Erhola M Hellings PW . Next-generation allergic rhinitis and its impact on asthma (aria) guidelines for allergic rhinitis based on grading of recommendations assessment, development and evaluation (GRADE) and real-world evidence. J Allergy Clin Immunol. (2020) 145:70–80.e3. doi: 10.1016/j.jaci.2019.06.049 31627910

[4] Torres MI Gil-Mata S Bognanni A Ferreira-da-Silva R Yepes-Nuñez JJ Lourenço-Silva N . Intranasal versus oral treatments for allergic rhinitis: a systematic review with meta-analysis. J Allergy Clin Immunol Pract. (2024) 12:3404–18. doi: 10.1016/j.jaip.2024.09.001 39251016

[5] Soe KK Krikeerati T Pheerapanyawaranun C Niyomnaitham S Phinyo P Thongngarm T. Comparative efficacy and acceptability of licensed dose intranasal corticosteroids for moderate-to-severe allergic rhinitis: a systematic review and network meta-analysis. Front Pharmacol. (2023) 14:1184552. doi: 10.3389/fphar.2023.1184552 37288109 PMC10242043

[6] Daley-Yates PT Larenas-Linnemann D Bhargave C Verma M. Intranasal corticosteroids: topical potency, systemic activity and therapeutic index. J Asthma Allergy. (2021) 14 1093–104. doi: 10.2147/JAA.S321332 34526783 PMC8436259

[7] De Vleeschhauwer F Casteels K Hoffman I Proesmans M Rochtus A. Systemic adverse events associated with locally administered corticosteroids. Children. (2024) 11 : doi: 10.3390/children11080951 PMC11353265 39201886

[8] Lu L Wen Q Zhang X Lv J Zhang L Liu L . Moxibustion as adjuvant therapy for preventing bone loss in postmenopausal women: protocol for a randomised controlled trial. BMJ Open. (2022) 12:e062677. doi: 10.1136/bmjopen-2022-062677 36523246 PMC9748964

[9] Cheng CH Chen LR Chen KH. Osteoporosis due to hormone imbalance: an overview of the effects of estrogen deficiency and glucocorticoid overuse on bone turnover. Int J Mol Sci. (2022) 23:1376. doi: 10.3390/ijms23031376 35163300 PMC8836058

[10] Hughes DE Dai A Tiffee JC Li HH Mundy GR Boyce BF. Estrogen promotes apoptosis of murine osteoclasts mediated by TGF-β. Nat Med. (1996) 2:1132–6. doi: 10.1038/nm1096-1132 8837613

[11] Kobza AO Herman D Papaioannou A Lau AN Adachi JD. Understanding and managing corticosteroid-induced osteoporosis. Open Access Rheumatol. (2021) 13:177–90. doi: 10.2147/OARRR.S282606 34239333 PMC8259736

[12] Chalitsios CV Shaw DE McKeever TM. Corticosteroids and bone health in people with asthma: a systematic review and meta-analysis. Resp Med. (2021) 181 106374. doi: 10.1016/j.rmed.2021.106374 33799052

[13] Hmamouchi I Paruk F Tabra S Maatallah K Bouziane A Abouqal R . Prevalence of glucocorticoid-induced osteoporosis among rheumatology patients in Africa: a systematic review and meta-analysis. Arch Osteoporos. (2023) 18:59. doi: 10.1007/s11657-023-01246-6 37129714

[14] Hofbauer LC Compston JE Saag KG Rauner M Tsourdi E. Glucocorticoid-induced osteoporosis: novel concepts and clinical implications. Lancet Diabetes Endo. (2025) 13:964–79. doi: 10.1016/S2213-8587(25)00251-7 41022095

[15] Watanabe H Sugiyama K Otsuji N Nakano K Arifuku H Wakayama T . Effect of inhaled corticosteroids on bone mineral density in patients with asthma. Asian Pac J Allergy. (2023) 41:45–52. doi: 10.12932/AP-191019-0663 32416663

[16] Israel E Banerjee TR Fitzmaurice GM Kotlov TV LaHive K LeBoff MS. Effects of inhaled glucocorticoids on bone density in premenopausal women. N Engl J Med. (2001) 345:941–7. doi: 10.1056/NEJMoa002304 11575285

[17] Loke YK Gilbert D Thavarajah M Blanco P Wilson AM. Bone mineral density and fracture risk with long-term use of inhaled corticosteroids in patients with asthma: systematic review and meta-analysis. BMJ Open. (2015) 5:e008554. doi: 10.1136/bmjopen-2015-008554 26603243 PMC4663435

[18] Fujita K Kasayama S Hashimoto J Nagasaka Y Nakano N Morimoto Y . Inhaled corticosteroids reduce bone mineral density in early postmenopausal but not premenopausal asthmatic women. J Bone Miner Res. (2001) 16:782–7. doi: 10.1359/jbmr.2001.16.4.782 11316007

[19] Rizzoli R Bischoff-Ferrari H Dawson-Hughes B Weaver C. Nutrition and bone health in women after the menopause. Womens Health (Lond). (2014) 10:599–608. doi: 10.2217/WHE.14.40 25482487

[20] Chen W Johnson KM FitzGerald JM Sadatsafavi M Leslie WD. Long-term effects of inhaled corticosteroids on bone mineral density in older women with asthma or COPD: a registry-based cohort study. Arch Osteoporos. (2018) 13:116. doi: 10.1007/s11657-018-0537-2 30374631

[21] Chiu KL Lee CC Chen CY. Evaluating the association of osteoporosis with inhaled corticosteroid use in chronic obstructive pulmonary disease in Taiwan. Sci Rep. (2021) 11:724. doi: 10.1038/s41598-020-80815-y 33436973 PMC7804267

[22] Pace WD Callen E Gaona-Villarreal G . Adverse outcomes associated with inhaled corticosteroid use in individuals with chronic obstructive pulmonary disease. Ann Fam Med. (2025) 23:127–35. doi: 10.1370/afm.240030 40127974 PMC11936369

[23] Wong CA Walsh LJ Smith CJ Shaikh A Yawn BP. Inhaled corticosteroid use and bone-mineral density in patients with asthma. Lancet. (2000) 355:1399–403. doi: 10.1016/S0140-6736(00)02138-3 10791523

[24] Chen M Fu W Xu H Liu CJ. Pathogenic mechanisms of glucocorticoid-induced osteoporosis. Cytokine Growth Factor Rev. (2023) 70:54–66. doi: 10.1016/j.cytogfr.2023.03.002 36906448 PMC10518688

[25] Fowler J Sowerby LJ. Using intranasal corticosteroids. Can Med Assoc J. (2021) 193:E47. doi: 10.1503/cmaj.201266 33431545 PMC7773038

[26] Konstandi M Johnson EO. Age-related modifications in CYP-dependent drug metabolism: role of stress. Front Endocrinol (Lausanne). (2023) 14:1143835. doi: 10.3389/fendo.2023.1143835 37293497 PMC10244505

[27] Ngcobo NN. Influence of ageing on the pharmacodynamics and pharmacokinetics of chronically administered medicines in geriatric patients: a review. Clin Pharmacokinet. (2025) 64:335–67. doi: 10.1007/s40262-024-01466-0 39798015 PMC11954733

[28] Pal China S Kalyanaraman H Zhuang S Cabriales JA Sah RL Pilz RB. Protein kinase G2 activation restores Wnt signaling and bone mass in glucocorticoid-induced osteoporosis in mice. JCI Insight. (2024) 9:e175089. doi: 10.1172/jci.insight.175089 38885330 PMC11383176

[29] Weinstein RS. Glucocorticoid-induced osteonecrosis. Endocrine. (2012) 41:183–90. doi: 10.1007/s12020-011-9580-0 22169965 PMC3712793

[30] Ilias I Milionis C Zoumakis E. An overview of glucocorticoid-induced osteoporosis. In:Feingold KR Adler RA Ahmed SF, editor. Endotext. South Dartmouth, MA: MDTextcom, Inc. (2022).

[31] Cho H Myung J Suh HS Kang HY. Antihistamine use and the risk of injurious falls or fracture in elderly patients: a systematic review and meta-analysis. Osteoporos Int. (2018) 29:2163–70. doi: 10.1007/s00198-018-4564-z 30046925

[32] American Geriatrics Society. American Geriatrics Society 2023 updated AGS Beers Criteria^®^ for potentially inappropriate medication use in older adults. J Am Geriatr Soc. (2023) 71:2052–81. doi: 10.1111/jgs.18372 37139824 PMC12478568

[33] Abrahamsen B Vestergaard P. Proton pump inhibitor use and fracture risk-effect modification by histamine H1 receptor blockade. Observational case-control study using national prescription data. Bone. (2013) 57:269–71. doi: 10.1016/j.bone.2013.08.013 23973557

[34] Giustina A Bouillon R Dawson-Hughes B Ebeling PR Lazaretti-Castro M Lips P . Vitamin D in the older population: a consensus statement. Endocrine. (2023) 79:31–44. doi: 10.1007/s12020-022-03208-3 36287374 PMC9607753

[35] Paccou J Yavropoulou MP Naciu AM Chandran M Messina OD Rolvien T . Prevention and treatment of glucocorticoid-induced osteoporosis in adults: recommendations from the European calcified tissue society. Eur J Endocrinol. (2024) 191:G1–17. doi: 10.1093/ejendo/lvae146 39556468

[36] Yuan C Liang Y Zhu K Xie W. Clinical efficacy of denosumab, teriparatide, and oral bisphosphonates in the prevention of glucocorticoid-induced osteoporosis: a systematic review and meta-analysis. J Orthop Surg Res. (2023) 18:447. doi: 10.1186/s13018-023-03920-4 37349750 PMC10286508

[37] Buckley LM Leib ES Cartularo KS Vacek PM Cooper SM. Calcium and vitamin D3 supplementation prevents bone loss in the spine secondary to low-dose corticosteroids in patients with rheumatoid arthritis. A randomized, double-blind, placebo-controlled trial. Ann Intern Med. (1996) 125:961–8. doi: 10.7326/0003-4819-125-12-199612150-00004 8967706

